# Correction: Green synthesis of silver nanoparticles using green tea leaf extract, characterization and evaluation of antimicrobial activity

**DOI:** 10.1039/d2na90063g

**Published:** 2022-08-10

**Authors:** Hiba Abbas Widatalla, Layla Fathi Yassin, Ayat Ahmed Alrasheid, Shimaa Abdelrahman Ahmed, Marvit Osman Widdatallah, Sahar Hussein Eltilib, Alaa Abdulmoneim Mohamed

**Affiliations:** Department of Pharmacology, Faculty of Pharmacy, University of Medical Sciences and Technology Khartoum Sudan hiba127@gmail.com; Department of Pharmaceutical Chemistry, Faculty of Pharmacy, University of Medical Sciences and Technology Khartoum Sudan; Department of Pharmacognosy, Faculty of Pharmacy, University of Medical Sciences and Technology Khartoum Sudan; Department of Clinical Pharmacy, Faculty of Pharmacy, University of Medical Sciences and Technology Khartoum Sudan

## Abstract

Correction for ‘Green synthesis of silver nanoparticles using green tea leaf extract, characterization and evaluation of antimicrobial activity’ by Hiba Abbas Widatalla *et al.*, *Nanoscale Adv.*, 2022, **4**, 911–915. https://doi.org/10.1039/D1NA00509J.

The authors regret mistakes in Fig. 3, where Fourier transform infrared data was depicted in a cartoon instead of presenting the raw data. The original IR data has been provided for both green tea leaf extract and silver nanoparticles, and is shown here.

**Fig. 1:**
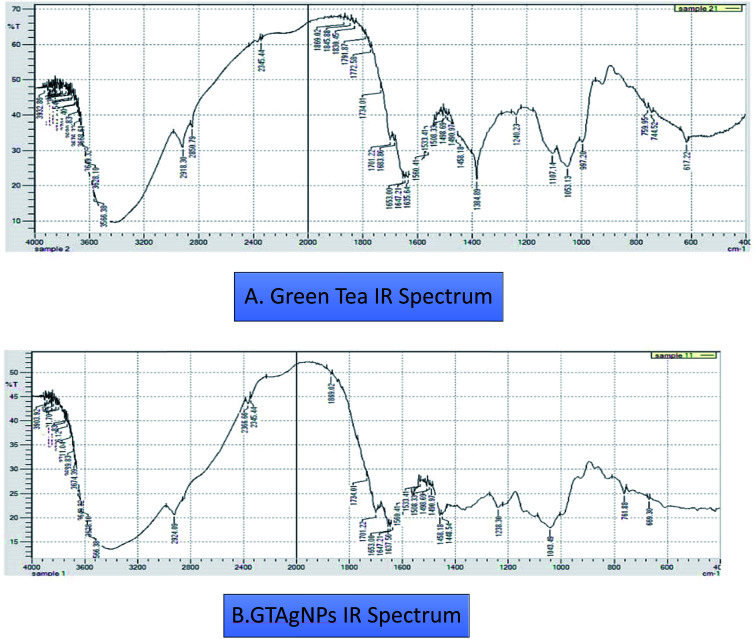
IR spectra of GT extract and GT AgNPs.

An independent expert has viewed the corrected images and has concluded that they are consistent with the discussions and conclusions presented.

The Royal Society of Chemistry apologises for these errors and any consequent inconvenience to authors and readers.

## Supplementary Material

